# Potential applications of next generation sequencing to the genomics of *Posidonia oceanica*

**DOI:** 10.3389/fpls.2012.00273

**Published:** 2012-12-05

**Authors:** Andrea Cavallini, Lucia Natali, Tommaso Giordani

**Affiliations:** Department of Agricultural, Food, and Environmental Sciences, University of PisaPisa, Italy

## Molecular studies on *Posidonia oceanica* are still limited

Despite of its paramount ecological importance in shallow shoreline habitats in the Mediterranean sea, basic knowledge of *Posidonia oceanica* genome is still limited, with regard to both functional and structural genomics.

Concerning protein-coding sequences, a survey of NCBI nucleotide database resulted in finding only 103 putative genes (on November 5, 2012). Of these, many are putative metallothionein (MT)- or aquaporin (AQP)-encoding genes.

These sequences have been extensively studied, since involved in the adaptation of *Posidonia* to endangered environments, at both physiological and genetic levels. Sequences encoding putative type2 MTs belong to a multigene family with at least two subgroups (Giordani et al., 2000; Cozza et al., 2006). Northern hybridizations indicated that MT transcripts accumulation is constitutive and seasonally regulated. However, MT transcripts accumulated after rhyzome harvesting and after 15 days of cultivation in an aquarium. As for animal MTs, transcripts accumulation was observed also after exposure to trace metals such as copper and cadmium. The two MT subgroups showed differences in their histological expression, i.e., in proliferative tissues or in lignified or suberized cells.

Concerning AQP gene family, two genes encoding AQPs of the plasmalemma and the tonoplast were isolated (Maestrini et al., 2004). Both genes were constitutively expressed in the leaves, with higher levels of transcripts in young than in differentiated leaf tissues. Variations of salt concentration in aquarium determined different AQPs transcript accumulation, suggesting that AQPs are involved in osmotic balance maintenance in seagrasses (Maestrini et al., 2004; Serra et al., 2011).

When surveying the *Posidonia* repetitive DNA, i.e., the intergenic, putatively non-coding portion of the genome, one can realize that this genome component is even less studied than coding portion. The 2C-genome size of *Posidonia* was established in 5–6 pg DNA (Cavallini et al., 1995; Koce et al., 2003), posing this species in the middle range of monocots, being genome much larger than in other seagrasses as *Cymodocea nodosa* (1.1 pg), *Zostera nolti* (1.5 pg), and *Z. marina* (1.2 pg) (Koce et al., 2003). Such differences are largely independent on chromosome number and hence on polyploidization: *P. oceanica* has 2*n* = 20 and *Zostera* species have 2*n* = 12 (Koce et al., 2003). *P. oceanica* genome has been characterized biochemically (Cavallini et al., 1995; Maestrini et al., 2002). At molecular level, only a few repetitive DNA sequences are found in the NCBI nucleotide database.

## Present status of *P. oceanica* genomics

During last years, the research for coding sequences in *P. oceanica* has proceeded through the production of both differential and EST libraries for obtaining sequences to be used in analyses of environmental adaptation of *Posidonia* meadows, especially associated with different light and temperature regimes and other potential stress-responsive gene networks. A SSH-cDNA library from a continuous meadow at two different depths (−5 and −25 m) produced 486 tentative unigenes, of which only 28 were common to both shallow and deep libraries (Dattolo et al., 2009). All genes were grouped in functional classes showing the highest differences between the two depths in primary metabolism, photosynthesis and stress defence genes.

Another recent study deals with the methylation status of *P. oceanica* chromosomal DNA, analysed by applying a Methylation-Sensitive Amplification Polymorphism technique, a modification of well-known AFLP procedure (Greco et al., 2012). This analysis allowed to identify a few loci that are differently methylated between Cd-treated and control plants.

In other experiments, a massive sequencing effort produced the first seagrass sequence database, Dr. Zompo, based on 14,597 ESTs obtained from *Z. marina* (9,412 ESTs) and *P. oceanica* (5,185) (Wissler et al., 2009). Dr. Zompo constitutes a valuable resource for the seagrass community, and is expected to grow in the near future, particularly with the recent initiation of the *Zostera* genome sequencing project.

*Zostera marina* sequencing initiative has started in 2010 under the guide of Dr. J. Olsen (University of Groningen) and it will greatly increase basic knowledge of seagrasses, allowing a deeper knowledge of seagrass biology and favoring studies in which analyses of genetic variability will be associated to the functional significance of differences.

Though sequencing of *Z. marina* genome will produce important consequences for all seagrasses, it is to be recalled that, from the genetic side, *P. oceanica* genome is much larger than that of *Z. marina* (around 5-fold). Major differences in genome size are generally related to polyploidization and/or to the amplification of non-coding, repetitive DNA, as for example centromeric and telomeric tandem repeats and, especially, transposable elements. Considering the different 2n-chromosome number of the two seagrass species (20 vs. 12), the large difference in genome size should be especially related to differences in repetitive DNA, though a cryptopolyploidy event cannot be excluded.

Repetitive DNA has long been considered as “selfish,” providing no adaptive benefit to the host genome. In the last years, repetitive DNA has shown to be essential for genome function. For example, transposons have a role in genome restructuring (Kazazian, 2000). Gene fragments rearranged by transposons could be the primary mechanism for generating new genes (Morgante et al., 2005). Other transposons effects on genome structure range from providing promoter and enhancer activity to modulating transcript elongation, contributing to pericentromeric and intercalary heterochromatin, supplying chromatin boundary signals for heterochromatin domains and hence playing a major architectonic role in higher order physical structuring of the nucleus (Von-Sternberg and Shapiro, 2005).

The influence of transposons on gene activity appears to be of special importance. Usually, phenotypic variation is conceived in terms of altered gene products produced by mutations in protein-coding sequences. However, other mechanisms generating variability do occur. For example, the organization of proteins can change without coding sequence modifications through changes in RNA splicing patterns via the integration of retroelements into introns (Nekrutenko and Li, 2001).

Transposon movements can change the regulatory formatting of conserved coding sequences. Such changes are even more important than those previously described, resulting in novel developmental patterns and new traits using the same assemblage of proteins and RNAs. In model organisms as the mouse and Arabidopsis, genetic studies on development have shown that retroelements play a role in the epigenetic settings of the genome, both globally, regulating chromatin organization in the nucleus (Van-Driel et al., 2003), and locally, as control elements of the expression of genes (Song et al., 2004). It appears that when the repetitive component of the genome changes, the epigenetic settings are modified, resulting in changes of phenotypic traits (Chong and Whitelaw, 2004).

## NGS techniques and perspectives for *Posidonia* genomics

Large size and high content of repetitive DNA elements are the major obstacles to genome sequencing: it is preferable to sequence model species with small genomes as *Zostera* and a concomitant similar initiative for *Posidonia* is not realistic at present.

Non-model species can be, however, conveniently explored using novel methods that imply next generation sequencing and bioinformatic analyses. Next generation sequencing apparently represents a step change and a starting point for genetical and biological research in the Twenty-first century, producing genomic sequences in parallel, at ever increasing speed and decreasing costs. So several Gigabases of data can be sequenced in a few weeks for a fraction of the costs of Sanger sequencing (Ansorge, 2009). NGS technologies offer novel, rapid ways for large, genome-wide characterization and profiling of DNA, mRNAs, small-RNAs, transcription factor regions, chromatin structure, and DNA methylation patterns.

For functional genomics studies, the next-generation technology allows the analysis of RNA transcripts by relatively short sequence tags, up to 150 nt for Illumina and 400–500 nt for 454, directly from each cDNA in the sample. Owing to the huge number of samples analysed simultaneously, sequence-based techniques can detect low abundance RNAs, small RNAs, or the presence of rare cells contained in the sample. Another advantage of this approach is that it does not require prior knowledge of the genome sequence.

Applications include precise quantification of RNA transcripts, measured through their sequence, without the probe hybridization employed in DNA chip techniques; identification and analysis of DNA regions that interact with regulatory proteins in functional regulation of gene expression; recovery and analysis of all components of a gene family.

Limitations of this technology are short read lengths, non-uniform confidence in base calling in sequence reads, particularly deteriorating 3′-sequence quality and generally lower reading accuracy in homopolar stretches of identical bases. Another limiting step is related to the huge amount of unorganized sequence data generated by these systems, that require the development of software and efficient computer algorithms.

## NGS techniques for studying *Posidonia* structural genomics

The contribution of repetitive DNA to genome structure and function has been studied in completely sequenced genomes. However, many questions remain about the distribution of repetitive sequences and the overall genome organization in plants with medium-large genomes, in which repetitive DNA is most abundant and presumably most important. Monocotyledons other than Graminaceae have in general been given little attention, and knowledge of the repetitive DNA in the genome evolution in seagrasses is totally lacking.

NGS technology can be conveniently applied for the identification of sequences present in many copies per genome, by producing of large numbers of short and randomly placed sequences and assembling them according to their sequence.

Actually, the major computational task in de novo assembling of millions of reads is represented by reads that map to multiple locations (that is, multi-reads). A combination of strategies have to be used in genome assembly for resolving problems caused by repetitive DNA, for example sequencing strategies that use fragment libraries of varying sizes. However, in searching repetitive DNA families, a smaller coverage can allow reducing redundancy of reads and hence favouring their assembly into contigs, obtaining repeat identification and reconstruction. Using low genome coverage, most of the contigs that are obtained do not represent specific genomic loci; instead, they are probably composed of reads derived from multiple copies of repetitive elements, thus representing consensus sequences of genomic repeats (Novák et al., 2010). Even though the exact sequence of this consensus does not necessarily occur in the genome, this representation of repetitive elements has been shown to be sufficiently accurate to enable amplification of the whole length repetitive elements using PCR (Swaminathan et al., 2007).

We have mentioned only a few of the potentialities of NGS technology to structural genomics of seagrasses. Such studies are expected to greatly improve the understanding of molecular physiology and adaptation mechanisms of these plants and represent an excellent opportunity for rapidly overcoming the present lack of knowledge.
